# Autologous Bone Marrow Cell Infusion for the Treatment of Decompensated Liver Cirrhosis Patients With Type 2 Diabetes Mellitus

**DOI:** 10.3389/fphys.2021.730797

**Published:** 2021-11-18

**Authors:** Baochi Liu, Mingrong Cheng, Lin Lang, Lei Li, Yanhui Si, Guangmian Wang

**Affiliations:** ^1^Department of Surgery, Shanghai Public Health Clinical Center, Fudan University, Shanghai, China; ^2^Department of Anorectal Surgery, Panzhou People’s Hospital, Guizhou, China; ^3^Shanghai New Hongqiao International Medical Center, Shanghai, China; ^4^Department of General Surgery, The First Affiliated Hospital of Zhengzhou University, Zhengzhou, China

**Keywords:** autologous bone marrow infusion, decompensated cirrhosis, type 2 diabetes, cell therapy, surgery-related complications

## Abstract

This study aimed to indicate whether autologous bone marrow cell infusion (ABMI) *via* the right omental vein (ROV) could have a regulatory effect on decompensated liver cirrhosis (DLC) patients with type 2 diabetes mellitus (T2DM). For this purpose, 24 DLC patients with T2DM were divided into observation group (*n*=14) and control group (*n*=10). Patients in the observation group were given ABMI through the ROV and right omental artery (ROA), and cases in the control group received ABMI through the ROV. At 1, 3, 6, and 12months after ABMI, it was revealed that the prothrombin time, the total bilirubin levels, and the amount of ascites were significantly lower, while the serum albumin levels in the two groups were markedly higher compared with those before ABMI (*p*<0.01), and there was no significant difference between the two groups at each time point (*p*>0.05). The fasting blood glucose and glycosylated hemoglobin levels at 6 and 12months after ABMI in the two groups significantly decreased compared with those before ABMI (*p*<0.05 or *p*<0.01), while the decreased levels in the observation group were more obvious than those in the control group at each time point (*p*<0.01). The amount of insulin in the observation group at 3, 6, and 12months after ABMI was significantly less than that before ABMI in the control group (*p*<0.01). In summary, ABMI showed a significant therapeutic efficacy for DLC patients with T2DM through ROV and ROA.

## Introduction

Cirrhosis is as a late stage of scarring (fibrosis) of the liver caused by liver diseases and conditions, such as hepatitis B virus (HBV)-induced cirrhosis, alcoholic cirrhosis, schistosomiasis-induced cirrhosis, metabolic cirrhosis, cholestasis cirrhosis, and cirrhosis of unknown etiology, and the most common type is HBV-induced cirrhosis in China (Chang et al., 2017). If no timely and effective antiviral treatment is provided for HBV-induced cirrhosis, it often progresses quickly to the stage of cirrhosis. In the late stage, serious complications, including decreased hepatic functional capacity, ascites, gastrointestinal bleeding, and even hepatic encephalopathy may occur. The liver is an important metabolic organ, and its metabolic activity is controlled by insulin and other metabolic hormones. Cirrhosis and diabetes are a two-way mutually-promoting process. Cirrhosis leads to abnormal glucose metabolism, and the elevated blood glucose level enhances damage to liver function and forms a vicious circle. Clinical treatment for cirrhosis is a highly complicated process (Xu et al., 2019; Michel et al., 2020), seriously influencing the quality of life and health of patients with cirrhosis.

The underlying mechanism of diabetes that contributes to cirrhosis is more complicated, and it is clinically manifested as insulin resistance (IR) and hyperinsulinemia. The underlying mechanism could be summarized as follows (Li et al., 2019): (I) In patients with liver cirrhosis, due to liver fatty degeneration and inflammatory damage, insulin receptors in the liver, muscles, pancreas, and other tissues decrease, and insulin affinity reduces, which in turn causes IR. At the same time, impaired liver function causes inactivation of liver glycogen synthase, hexokinase, glucokinase, growth hormone, and other obstacles, thereby leading to an abnormal glycogen synthesis and the increased gluconeogenesis (Kumar, 2018); (II) The decreased hepatic insulin clearance may result in peripheral hyperinsulinemia, because the liver function in patients with liver cirrhosis reduces and portosystemic shunts increase. Due to the long-term stimulation of hyperglycemia, secretion of compensatory insulin from pancreatic islet β cells increases in the early stage, and dysfunction of pancreatic islet β cells may occur in the later stage, and then, develops into diabetes (Grancini et al., 2015); and (III) In addition, it may also be associated with hepatitis viruses (types C and D) and toxic effects, including immune complexes, degeneration of pancreatic islet β cells, electrolyte imbalance, leptin resistance, genetic inheritance, and other factors (Elkrief et al., 2016). Type 2 diabetes mellitus (T2DM) could promote the development of hepatitis cirrhosis, increase the incidence of associated complications, and enhance the occurrence of liver cancer (Su et al., 2015). Diabetes is an independent risk factor for primary liver cancer, and is associated with HBV, hepatitis C virus (HCV), and alcoholic liver disease, leading to increase the risk of liver cancer (Dankner et al., 2016). Therefore, how to effectively block the vicious circle between cirrhosis and diabetes is the key to the treatment of diabetic patients with cirrhosis.

The most appropriate treatment strategy for decompensated liver cirrhosis (DLC) is orthotopic liver transplantation. However, due to the shortage of donors, complicated surgery, remarkable expenses, and the long-term use of immunosuppressants, its clinical application is limited. Cell therapy includes hepatocyte transplantation and stem cell therapy, both of which are theoretically feasible (Sheykhhasan, 2019). Hepatocytes have the ability to regenerate, and a number of scholars theoretically pointed out that hepatocyte transplantation should be able to restore the liver function after liver cirrhosis treatment. However, hepatocytes’ viability under *in vitro* cryopreservation decreases, which limits their clinical application to a certain extent (Komolkriengkrai et al., 2019). Stem cells are a type of cells with multiple differentiation potentials, and can differentiate into different cells in various environments. With the rapid development of stem cell research, stem cell transplantation has markedly attracted clinicians’ attention to treat cirrhosis (Memon and Abdelalim, 2020). Bone marrow-derived stem cells (BMSCs) are the main extrahepatic source of hepatocytes. BMSCs can differentiate into hepatic stem cells and hepatocytes under specific circumstances, and then, repair the liver function (Bihari et al., 2016). In our previous study, we used autologous bone marrow cell infusion (ABMI) *via* an infusion port through the right omental vein (ROV) to achieve a significant therapeutic efficacy (Liu et al., 2013). A number of patients even stopped taking oral hypoglycemic drugs. Although the treatment of liver cirrhosis has been found to have a certain improvement effect on diabetes, if we inject autologous bone marrow through the right omental artery (ROA) directly on the pancreas, whether it is more effective or not requires further research. Based on the above-mentioned clinical findings, our team, in this research, embedded the infusion port through the ROA and ROV, collected autologous bone marrow (ABM), performed infusion *via* the ROA and ROV, and attempted to indicate whether it could further improve the treatment efficacy for DLC patients with T2DM.

## Materials and Methods

### Study Subjects

A total of 24 DLC patients with T2DM, who were admitted to our hospital from January 2015 to December 2018, were selected for the retrospective study [male (18) vs. female (6); range of age, 31–65years old; median age, 46.96±6.87years old]. There were 14 cases of HBV-induced cirrhosis, four cases of alcoholic cirrhosis, four cases of HCV-induced cirrhosis, and two cases of schistosomiasis-induced cirrhosis. According to the Child-Pugh scoring system, there were 18 cases of grade B and six cases of grade C. The research has been carried out in accordance with the Declaration of Helsinki, and all patients signed the written informed consent form prior to starting the study.

#### Inclusion Criteria

Computed tomography (CT) scans showed that the volume of each lobe of the liver was significantly reduced, as well as displaying a cirrhotic liver with an irregular shape and splenomegaly; occurrence of peritoneal effusion; satisfying the diagnostic criteria for DLC and T2DM.

#### Exclusion Criteria

Patients’ age<18years old; pregnant and lactating women; malignant tumors in the liver or other organs; patients with spontaneous bacterial peritonitis or gastrointestinal bleeding; patients with acute heart failure, respiratory insufficiency, or other diseases who were intolerant to treatment; patients who underwent hormone therapy; patients with intellectual disabilities or mental disorders who were unable to participate in surgery and follow-up.

### Treatment Methods

#### General Treatments

Cirrhotic patients with T2DM received conventional liver protection, diuresis and insulin, or hypoglycemic drugs, and antiviral treatment for hepatitis viruses. Those patients were routinely prepared for surgery, and the liver protection and diuresis, insulin, and other treatments were also provided before and after surgery.

#### Grouping

According to different ABMI routes, they were divided into observation group (*n*=14) and control group (*n*=10), this study was non-randomized and non-blinded. Patients in the observation group were intubated and implanted *via* the ROA and ROV, respectively. Cases in the control group were infused through the ROV. Among them, there were seven males and three females in the control group, aged from 31 to 65years old, with an average age of 46.10±8.91years, the course of disease was 1–18years, and the average course of disease was 8.93±3.72years, body mass index (BMI) was 21.63±1.26kg/m^2^, three cases with hypertension; in the observation group, there were 11 males and three females, and the age was 38–59years old, average age was 47.57±5.24years old, disease course was 1~15years, average disease course was 8.32±2.94years, and BMI was 21.82±1.32kg/m^2^, five cases with hypertension. There was no statistically significant difference between the two groups in age, gender, course of disease, BMI, with hypertension, liver function indicators, and other baseline data (*p*>0.05), and they were comparable.

#### Establishment of Input Channels

Surgery was performed under general anesthesia or local anesthesia, a small incision was made in the upper abdomen, the suction negative pressure was used to partly release the ascites in patients with large ascites, the anterior gastric wall was lifted, and the gastric omental blood vessels were found.

#### Extraction and Infusion of ABM

Patients were fasted at 6h before surgery. During surgery, 40–80ml ABM was extracted from the anterior superior iliac spine, and then, the infusion port was percutaneously punctured as buried under the skin of the upper abdomen. After that, 40ml ABM was infused *via* ROV in the control group and *via* ROV (40ml ABM) and ROA (40ml ABM) in the observation group, respectively, and 5ml heparin saline was finally injected into the infusion port to prevent blood clotting. At 1 and 3months after surgery, 40 or 80ml ABM was infused again through the infusion port.

#### Extraction of Blood Samples

EDTA anti-condensing pipe for bloodocyte analysis specimens, vacuum negative pressure non-anti-condensate pipe for the liver and kidney function detection specimens and containing sodium citrate pipe for coagulation function specimens was used. First, 5ml blood was drawn using a median cubital vein before surgery, and at 1, 3, 6, and 12months after ABMI, placed into an anticoagulation tube, and allowed to stand at room temperature for 30min. The sample was centrifuged by Tengying Machinery Manufacturing Co., Ltd. (Zhangjiagang, China) at 3,000rpm for 15min with a centrifugal radius of 9cm. The supernatant was collected and stored in a refrigerator (−80°C).

#### Biochemical Analysis

According to the manufacturer’s instructions, the AU5800 automatic biochemical analyzer (Beckman Coulter Inc., Brea, CA, United States) was used to detect the serum levels of total bilirubin (TB), albumin (ALB), and glycosylated hemoglobin (HbA1c). The prothrombin time (PT) was measured by the CA-500 automatic coagulation analyzer (Sysmex Corporation, Kobe, Japan); the fasting blood glucose (FBG) levels were detected with a fast blood glucose meter (OMRON Corporation, Kyoto, Japan).

#### Determination of Ascites

In 1996, Inadomi et al. (1996) reported a method to measure the volume of ascites using ultrasound. The ascites was defined as an abnormal amount of intraperitoneal fluid. Two factors (abdominal circumference and maximum depth of ascites) were measured. To measure these two factors, patients were asked to lie on their back and lie on their stomach, the abdominal circumference (*C*) around the umbilicus was determined, and then, patients were asked to lie on the prone position. The ultrasonic probe measured the maximum depth of ascites at the umbilical circumference (d), that is, the maximum vertical distance from the interface of the floating intestinal loop to the probe. The above-mentioned data were imported into the following formula to calculate the volume of ascites (*V*) as follows: *V*=1/3 [πd^2^ (3r-d)], where “*r*” is the radius of the abdominal cavity (*r*=*C*/2π).

#### Statistical Analysis

The SPSS 19.0 software (IBM Corp., Armonk, NY, United States) was used to statistically process the data. Shapiro-wilk test was used for normal distribution. The measured data conforming to the normal distribution were presented as mean±SD. The data of observation group and control group were compared using the *t*-test; the statistical value was expressed by the *t* value. The abnormally distributed data were expressed as the median and interquartile range (P25, P75), and analyzed using the Mann-Whitney U test, the statistical value was expressed by the *z* value. One-way ANOVA analysis was performed in Multi-group comparison, multiple two or two more compared to use Least Significant Difference. Categorical data were analyzed by the Chi-square test. *p*<0.05 was considered statistically significant.

## Results

### Surgery-Associated Complications

In the control group, two cases of wound congestion were implanted in the abdomen postoperatively, four cases of wound congestion were implanted in the abdomen in the observation group, and wound congestion in the two groups was gradually absorbed around 10days after surgery.

### Changes of Hepatic Synthesis and Secretion Function

**Figure 1:**
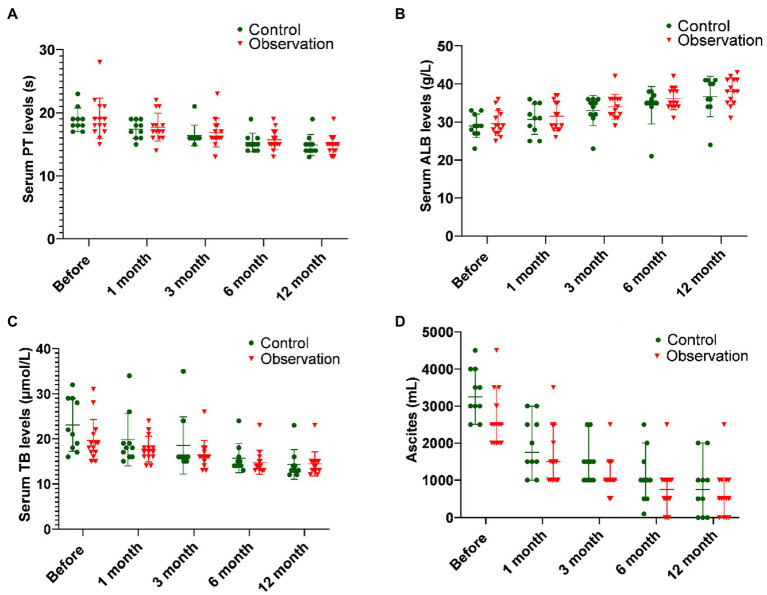
Comparison of liver synthesis function and secretion function in DLC patients with T2DM before and after ABMI. **(A)** The PT before and after ABMI. **(B)** The serum ALB levels before and after ABMI. **(C)** The serum TB levels before and after ABMI. **(D)** The amount of ascites in the two groups before and after ABMI. DLC, decompensated liver cirrhosis; T2DM, type 2 diabetes mellitus; ABMI, autologous bone marrow infusion; PT, prothrombin time; ALB, albumin; and TB, total bilirubin.

As shown in Figure 1A and Table 1, there was no significant difference in PT before ABMI and at each time point after ABMI between the two groups (*p*>0.05), PT in the two groups at 1, 3, 6, and 12months after ABMI significantly decreased compared with that before ABMI (*p*<0.01), PT in the two groups at 6 and 12months after ABMI significantly decreased compared with that at 1month (*p*<0.01or *p*<0.05), and PT in the two groups at 12months after ABMI significantly decreased compared with that at 3month (*p*<0.05).

**Table 1:** The prothrombin time (PT) in the two groups before and after autologous bone marrow cell infusion (ABMI; mean±SD, s).

Group	Before	1month	*t/p*	3months	*t/p*	6months	*t/p*	12months	*t/p*
Control	18.90±1.85	17.40±1.43	3.503/0.000	16.40±1.65	7.319/0.000	15.30±1.49	9.000/0.000	14.90±1.66	18.974/0.000
Observation	19.14±3.20	17.71±2.23	3.150/0.008	16.86±2.28	5.551/0.000	15.71±1.59	6.213/0.000	14.93±1.49	8.012/0.000
*T*	0.214	0.390		0.540		0.645		0.044	
*p*	0.832	0.700		0.595		0.526		0.965	

PT, prothrombin time; ABMI, autologous bone marrow infusion. *t/p* means the above *t* means the value of *t* and the lower *p* means the value of *p*.

It could be seen from Figure 1 and Table 2 that the serum ALB levels before ABMI and at each time point after ABMI were not significantly different in the two groups, the serum ALB levels in the two groups at 1, 3, 6, and 12months after ABMI were markedly higher than those before ABMI (*p*<0.01), the serum ALB levels in the control group at 12months after ABMI were markedly higher than those at 1month, the serum ALB levels in the observation group at 6, and 12months after ABMI were markedly higher than those at 1month, and the serum ALB levels in the control group at 12months after ABMI were markedly higher than those at 3months.

**Table 2:** The serum ALB levels in the two groups before and after ABMI (mean±SD, g/L).

Group	Before	1month	*t/p*	3months	*t/p*	6months	*t/p*	12months	*t/p*
Control	29.00±3.09	30.70±3.91	2.940/0.016	33.00±3.97	6.325/0.000	34.40±4.90	5.449/0.000	36.70±5.31	7.229/0.000
Observation	29.64±3.32	31.50±3.84	3.627/0.000	34.00±3.28	8.214/0.000	36.14±2.91	10.835/0.000	38.00±3.46	10.788/0.000
*T*	0.481	0.496		0.675		0.787		0.727	
*p*	0.635	0.625		0.507		0.286		0.457	

ALB, albumin; ABMI, autologous bone marrow infusion. *t/p* means the above *t* means the value of *t* and the lower *p* means the value of *p*.

As illustrated in Figure 1C and Table 3, there was no significant difference in the serum TB levels between the two groups before ABMI and at each time point after ABMI (*p*>0.05), the serum TB levels at 1, 3, 6, and 12months after ABMI were noticeably reduced in the two groups compared with those before ABMI (*p*<0.01), the serum TB levels in control group at 12months after ABMI were noticeably reduced compared with those at 1months (*p*<0.05), and the serum TB levels in observation group at 6, and 12months after ABMI were noticeably reduced compared with those at 1months (*p*<0.01).

**Table 3:** The serum TB levels in the two groups before and after ABMI (mean±SD, μmol/L).

Group	Before	1month	*t/p*	3months	*t/p*	6months	*t/p*	12months	*t/p*
Control	23.10±5.86	19.80±5.85	2.386/0.041	18.50±6.37	2.953/0.016	15.70±3.24	5.543/0.000	14.30±3.27	6.300/0.000
Observation	19.64±4.67	17.71±2.87	3.280/0.006	16.43±3.16	3.079/0.009	14.79±2.67	4.342/0.000	14.43±2.71	5.662/0.000
*T*	0.481	0.496		0.675		1.093		0.727	
*p*	0.635	0.625		0.507		0.286		0.475	

TB, total bilirubin; ABMI, autologous bone marrow infusion. *t/p* means the above *t* means the value of *t* and the lower *p* means the value of *p*.

It could be seen from Figure 1D and Table 4 that there was no significant difference in the amount of ascites before ABMI and at each time point after ABMI between the two groups (*p*>0.05), the amount of ascites at 1, 3, 6, and 12months after ABMI significantly decreased in the two groups compared with that before ABMI (*p*<0.05 or<0.01), the amount of ascites at 6, and 12months after ABMI significantly decreased in the two groups compared with that at 1month (*p*<0.01), and the amount of ascites at 12months after ABMI significantly decreased in the control groups compared with that at 3month (*p*<0.05).

**Table 4:** The amount of ascites in the two groups before and after ABMI [*M* (*P*_25_, *P*_75_), ml].

Group	Before	1month	*z/p*	3months	*z/p*	6months	*z/p*	12months	*z/p*
Control	3,250 (2,875~4,000)	1,750 (1,375~2,625)	2.825/0.000	1,500 (1,000~1750)	2.814/0.005	1,000 (500~1,625)	2.821/0.005	750 (0~1,250)	2.809/0.005
Observation	2,500 (2,375~3,750)	1,500 (1,000~2,125)	3.347/0.001	1,000 (1,000~1,500)	3.326/0.001	750 (375~1,000)	3.367/0.001	500 (0~1,000)	3.317/0.001
*Z*	1.073	1.056		1.604		1.270		0.290	
*p*	0.283	0.291		0.148		0.204		0.772	

ABMI, autologous bone marrow infusion. *z/p* means the above *z* means the value of *z* and the lower *p* means the value of *p*.

### Changes in the Function of Islet β Cells Before and After ABMI

**Figure 2:**
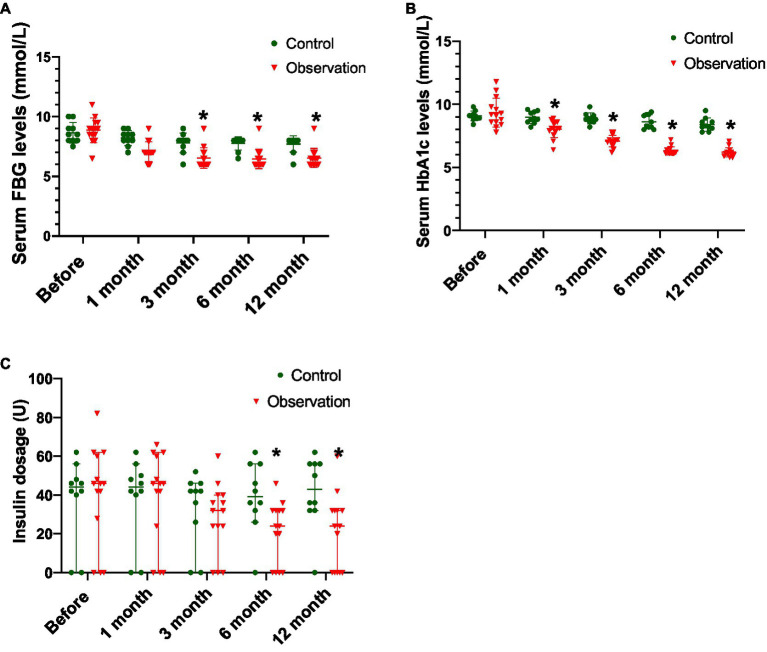
Comparison of disease control in DLC patients with T2DM before and after ABMI. **(A)** The serum FBG levels in the two groups before and after ABMI. **(B)** The serum HbA1c levels in the two groups before and after ABMI. **(C)** The insulin dosage in the two groups before and after ABMI. DLC, decompensated liver cirrhosis; T2DM, type 2 diabetes mellitus; ABMI, autologous bone marrow infusion; FBG, fasting blood glucose; and HbA1c, glycosylated hemoglobin; Compared with control group, ^*^p<0.01.

As shown in Table 5 and Figure 2A, there was no significant difference in the serum FBG levels between the two groups before ABMI (*p*>0.05), and the serum FBG levels at 1 (observation group only), 3, 6, and 12months after ABMI in the two groups significantly decreased compared with those before ABMI (*p*<0.05 or<0.01), in which the decreased levels in the observation group were more significantly obvious than those in the control group at each time point after ABMI (*p*<0.01).

**Table 5:** The serum FBG levels in the two groups before and after ABMI (mean±SD, mmol/L).

Group	Before	1month	*t/p*	3months	*t/p*	6months	*t/p*	12months	*t/p*
Control	8.65±0.85	7.35±2.66	1.868/0.095	7.80±0.82	2.940/0.016	7.75±0.54	3.515/0.007	7.70±0.67	3.475/0.007
Observation	8.86±1.05	7.07±0.89	6.250/0.000	6.536±0.87	7.038/0.000	6.46±0.82	7.697/0.000	6.54±0.87	7.878/0.000
*T*	0.834	0.371		3.600		4.322		3.756	
*p*	0.611	0.741		0.002		0.000		0.001	

FBG, fasting blood glucose; ABMI, autologous bone marrow infusion. *t/p* means the above *t* means the value of *t* and the lower *p* means the value of *p*.

From Table 6 and Figure 2B, there was no significant difference in the serum HbA1c levels between the two groups before ABMI (*p*>0.05), the serum HbA1c levels in the control group at 6 and 12months after ABMI were markedly lower than those before ABMI (*p*<0.01), and the serum HbA1c levels in the control group at 12months after ABMI were markedly lower than those at 1 and 3months (*p*<0.01).

**Table 6:** The serum HbA1c levels in the two groups before and after ABMI (mean±SD, mmol/L).

Group	Before	1month	*t/p*	3months	*t/p*	6months	*t/p*	12months	*t/p*
Control	9.08±0.38	8.96±0.47	1.024/0.333	8.89±0.43	1.812/0.103	8.62±0.55	3.977/0.003	8.40±0.52	5.540/0.000
Observation	9.35±1.14	8.04±0.68	4.990/0.000	7.09±0.46	8.587/0.000	6.31±0.32	10.153/0.000	6.20±0.36	11.717/0.000
*T*	0.714	3.674		9.730		11.850		12.249	
*p*	0.483	0.001		0.000		0.000		0.000	

HbA1c, glycosylated hemoglobin; ABMI, autologous bone marrow infusion. *t/p* means the above *t* means the value of *t* and the lower *p* means the value of *p*.

Besides, the serum HbA1c levels in the observation group at 1, 3, 6, and 12months after ABMI were significantly lower than those before ABMI (*p*<0.01), the serum HbA1c levels in the observation group at 3, 6, and 12months after ABMI were significantly lower than those at 1month (*p*<0.01), the serum HbA1c levels in the observation group at 6, and 12months after ABMI were significantly lower than those at 3months (*p*<0.01), and the decreased levels in the observation group at different time points after ABMI were more significantly obvious than those in the control group (*p*<0.01).

From Table 7 and Figure 2C, there was no significant difference in the amount of insulin before ABMI between the two groups (*p*>0.05), the amount of insulin in the observation group at 3, 6, and 12months after ABMI was significantly less than that before ABMI in the control group (*p*<0.01), and the amount of insulin in the observation group at 6, and 12months after ABMI was significantly less than that at 6, and 1month (*p*<0.01).

**Table 7:** The insulin dosage in the two groups before and after ABMI [*M* (P25, P75), U].

Group	Before	1month	*z/p*	3months	*z/p*	6months	*z/p*	12months	*z/p*
Control	44 (30~50)	46 (35~61)	1.00/0.317	44 (30~52)	1.841/0.066	42 (20~46)	0.000/1.000	39 (31~56)	0.676/0.499
Observation	43 (32~56)	46 (33~61)	1.342/0.180	32 (24~40)	2.810/0.005	24 (10~32)	2.936/0.003	24 (0~32)	2.668/0.008
*Z*	0.659	0.500		1.124		2.442		2.393	
*p*	0.510	0.617		0.261		0.015		0.017	

ABMI, autologous bone marrow infusion. *z/p* means the above *z* means the value of *z* and the lower *p* means the value of *p*.

## Discussion

Decompensated liver cirrhosis is defined as an acute deterioration in liver function in patients with cirrhosis and is characterized by jaundice, ascites, hepatic encephalopathy, hepatorenal syndrome, or variceal hemorrhage, while no effective therapy has been presented for DLC yet (Bernardi and Caraceni, 2018). Although liver transplantation has shown to be clinically significant for liver cirrhosis, its clinical application has been limited due to the serious shortage of donors and remarkable expenses. In recent years, several studies have reported that BMSCs could be effective for the repair of liver function (Liu et al., 2014; Pan et al., 2014), and this finding has also been confirmed in animal models. In addition, a limited number of clinical studies showed that transplantation of BMSCs could quickly improve the liver function without obvious adverse effects (Wu et al., 2019; Sun et al., 2020), indicating the lack of a multi-center and systematic research and the absence of an effective treatment plan for liver cirrhosis (Esmaeilzadeh et al., 2019). In the present study, the levels of ALB, TB, PT, and ascites were significantly elevated in the two groups at 1month after treatment, suggesting that the hepatic synthesis and secretion function were significantly improved. With the elevation of ALB level, the colloid osmotic pressure increased, the degree of liver cirrhosis and portal pressure decreased, and the amount of ascites was gradually reduced. After 3–4 times of ABMI through the portal vein, the liver function was significantly improved after 1year of follow-up, and the liver function partly returned to normal. It is well known that there are a variety of stem cells and cytokines in the bone marrow. Treatment *via* ABMI can promote the repair of liver function in patients with cirrhosis. Stem cells have the potential to differentiate into a variety of cells affected by certain factors in different microenvironments, and they can also produce certain cytokines to promote the repair of damaged cells (Zhang et al., 2012; Liu et al., 2013). The treatment mechanism of BMSCs for cirrhosis was previously described as follows (Zhang and Wang, 2013): (I) On the one hand, the hepatocyte-like BMSCs could be transferred into the liver parenchyma under the stimulation of the microenvironment; on the other hand, they could differentiate into liver oval cells, and they could be directly differentiated into liver parenchymal cells, hepatic stellate cells, and myofibroblasts. The hepatic oval cells were discovered by Farber when he was studying the pathology of liver tumors. They were named as oval cells because they were oval-shaped cells. There are few oval cells in the adult liver, which are mainly distributed around the portal vein at rest. When the liver is stimulated by pathogenic factors, the existing oval cells and newly formed oval cells can be differentiated into hepatocytes, bile duct cells, etc., due to the changes in the microenvironment and stimulation of cytokines (Farber, 1956); (II) Improving the microenvironment through paracrine when BMSCs can be entered the liver, whether there is cell differentiation or anti-liver fibrosis, they are inseparable from various cytokines and inflammatory mediators. The cytokines and inflammatory mediators could activate liver progenitor cells, thereby inhibiting the activation of stellate cells and promoting their apoptosis, as well as inhibiting the proliferation and migration of inflammatory cells at the injury site (Suh et al., 2012). In the present study, it was shown that the liver function was improved in DLC patients with T2DM, while the difference between the two groups was not statistically significant, indicating that the ABMI through the ROA did not improve the liver function.

Under fasting conditions, the liver plays a major role in generating glucose as a fuel for other tissues, such as brain, red blood cells, and muscles. Normally, stored glycogen is critical for maintaining glucose homeostasis in mammals during an overnight fasting period. Therefore, there is an inseparable association between the occurrence of liver disease and glucose metabolism disorders. Globally, the incidence of diabetes was estimated about 1% in healthy individuals (Quist-Therson et al., 2020). The incidence of diabetes was significantly elevated in patients with chronic liver dysfunction, especially in patients with liver cirrhosis, in which more than 80% of patients had impaired glucose tolerance, and 40–50% of patients were at a high risk of developing diabetes (Petit et al., 2014; Grancini et al., 2015). Therefore, it is vital for patients with hepatogenic diabetes to receive clinical attention and proactive treatment (Zhang et al., 2019). In the current study, ABMI was performed only *via* the ROV. After 1month, the liver function improved, and the serum FBG and HbA1c levels decreased, which is consistent with the finding of a previous research, in which a successful stem cell therapy for chronic liver cell failure patients could improve T2DM by increasing IR (Haydara et al., 2019). However, with the continuously infusion of ABM through the portal vein, as the liver function continued to improve, the serum FBG and HbA1c levels did not significantly change, while more significant changes were observed in the observation group, suggesting that the main reason for increasing the serum FBG levels was not related to the liver function, and pancreatic islet dysfunction in patients with liver cirrhosis and diabetes is a more important factor. The results of the current research revealed that the amount of insulin used in the observation group was significantly lower than that in the control group before ABMI, indicating that ABMI *via* ROV did not significantly reduce the amount of insulin, implying that it did not improve the physiological function of islet β cells. ABM was used to infuse through the ROA, BMSCs could enter the pancreas through the ROA, BMSCs might be transformed into islet β cells, or secrete certain cytokines to promote the repair and reconstruction of damaged islet β cells, and the regulatory function of blood glucose could be improved. If the ABMI was infused only through the portal vein, some stem cells might return to the right atrium and right ventricle through the hepatic vein. After the pulmonary circulation, they could enter the systemic circulation, and a limited number of stem cells might enter the pancreas. Because the number of stem cells was extremely small, the performance of islet β cells slightly improved, while ABMI through the ROA significantly improved the performance of islet β cells compared with the ROV infusion alone. This also suggested that the infusion of BMSCs into the liver tissue with cirrhosis could repair the damaged hepatocytes, leading to improve the liver function, and the infusion of BMSCs into the pancreas could repair the damaged islet β cells. The microenvironment with the cell damage in different organs could promote the transformation of BMSCs into different cells or secrete certain factors to promote the functional recovery of injured organs.

## Conclusion

In summary, the ABMI *via* ROV and ROA showed noticeable effects on the treatment of DLC patients with T2DM. The ABMI *via* the ROV did not significantly improve diabetes, while ABMI *via* the ROA did not remarkably improve the liver function.

## Data Availability Statement

The original contributions presented in the study are included in the article/supplementary material, further inquiries can be directed to the corresponding author.

## Ethics Statement

The studies involving human participants were reviewed and approved by Shanghai Public Health Clinical Center Ethics Committee. The patients/participants provided their written informed consent to participate in this study.

## Author Contributions

BL conceived and designed the experiments. MC and LLa performed this experimental work and analyzed the data. LLi, YS, and GW participated in the experiments. All authors contributed to the article and approved the submitted version.

## Funding

This study was financially supported by Shanghai Shenkang Hospital Development Center (Grant No. SHDC12016129) and Scientific Research Project of Shanghai Municipal Health Commission (Grant No. 201940430).

## Conflict of Interest

The authors declare that the research was conducted in the absence of any commercial or financial relationships that could be construed as a potential conflict of interest.

## Publisher’s Note

All claims expressed in this article are solely those of the authors and do not necessarily represent those of their affiliated organizations, or those of the publisher, the editors and the reviewers. Any product that may be evaluated in this article, or claim that may be made by its manufacturer, is not guaranteed or endorsed by the publisher.

## Abbreviations

IR: insulin resistance
DLC: decompensated liver cirrhosis
BMSCs: bone marrow-derived stem cells
ABMI: autologous bone marrow infusion
HIV: human immunodeficiency virus
ROV: right omental vein
ROA: right omental artery
T2DM: type 2 diabetes mellitus
TB: total bilirubin
ALB: albumin
HbA1c: glycosylated hemoglobin
PT: prothrombin time
FBG: fasting blood glucose
SD: standard deviation
